# Metabolic acidosis caused by concomitant use of paracetamol (acetaminophen) and flucloxacillin? A case report and a retrospective study

**DOI:** 10.1007/s00228-017-2311-6

**Published:** 2017-08-07

**Authors:** J. K. Berbee, L. A. Lammers, C. T. P. Krediet, J. C. Fischer, E. M. Kemper

**Affiliations:** 1 Department of Hospital Pharmacy, Academic Medical Centre, Meibergdreef 9, 1105 AZ Amsterdam, the Netherlands; 2 Department of Internal Medicine, Academic Medical Centre, Amsterdam, the Netherlands; 3 Department of Clinical Chemistry, Academic Medical Centre, Amsterdam, the Netherlands

**Keywords:** Anion gap, Acidosis, Paracetamol, Flucloxacillin, Interaction, 5-oxoproline

## Abstract

**Purpose:**

A patient was identified with severe metabolic acidosis, a high anion gap and 5-oxoproline accumulation, probably caused by the simultaneous use of paracetamol (acetaminophen) and flucloxacillin. We wanted to investigate the necessity to control the interaction between both drugs with an automatic alert system.

**Methods:**

To investigate the relevance of the interaction of paracetamol and flucloxacillin, a retrospective study was conducted. Data on paracetamol and flucloxacillin prescriptions and laboratory data (pH, Na^+^, HCO_3_^−^, Cl^−^, albumin and 5-oxoproline levels) were combined to assess the prevalence of acidosis, calculate the anion gap and analyse 5-oxoproline levels in clinically admitted patients using both drugs simultaneously.

**Results:**

In the 2-year study period, approximately 53,000 admissions took place in our hospital. One thousand and fifty-seven patients used paracetamol and flucloxacillin simultaneously, of which 51 patients (4.8%) had a serum pH ≤ 7.35. One patient, the same patient as presented in the case report, had a high anion gap and a toxic level of 5-oxoproline.

**Conclusion:**

The prevalence of metabolic acidosis is very low and the only patient identified with the interaction was recognised during normal clinical care. We conclude that automatic alerts based on simultaneous use of paracetamol and flucloxacillin will generate too many signals. To recognise patients earlier and prevent severe outcomes, a warning system (clinical rule) based on paracetamol, flucloxacillin and pH measurement may be helpful. Early calculation of the anion gap can narrow the differential diagnosis of patients with metabolic acidosis and measurement of 5-oxoproline can explain acidosis due the interaction of paracetamol and flucloxacillin.

**Electronic supplementary material:**

The online version of this article (doi:10.1007/s00228-017-2311-6) contains supplementary material, which is available to authorized users.

## Introduction

Metabolic acidosis in patients admitted to the hospital is associated with morbidity and mortality. It is necessary to recognise the cause of the acidosis in order to intervene quickly and to treat effectively. On basis of multiple case reports, a specific cause of metabolic acidosis in hospital patients has been recognised, i.e. increased levels of 5-oxoproline (pyroglutamic acid) due to the interaction between paracetamol (acetaminophen) and flucloxacillin [1–7].

**Fig. 1:**
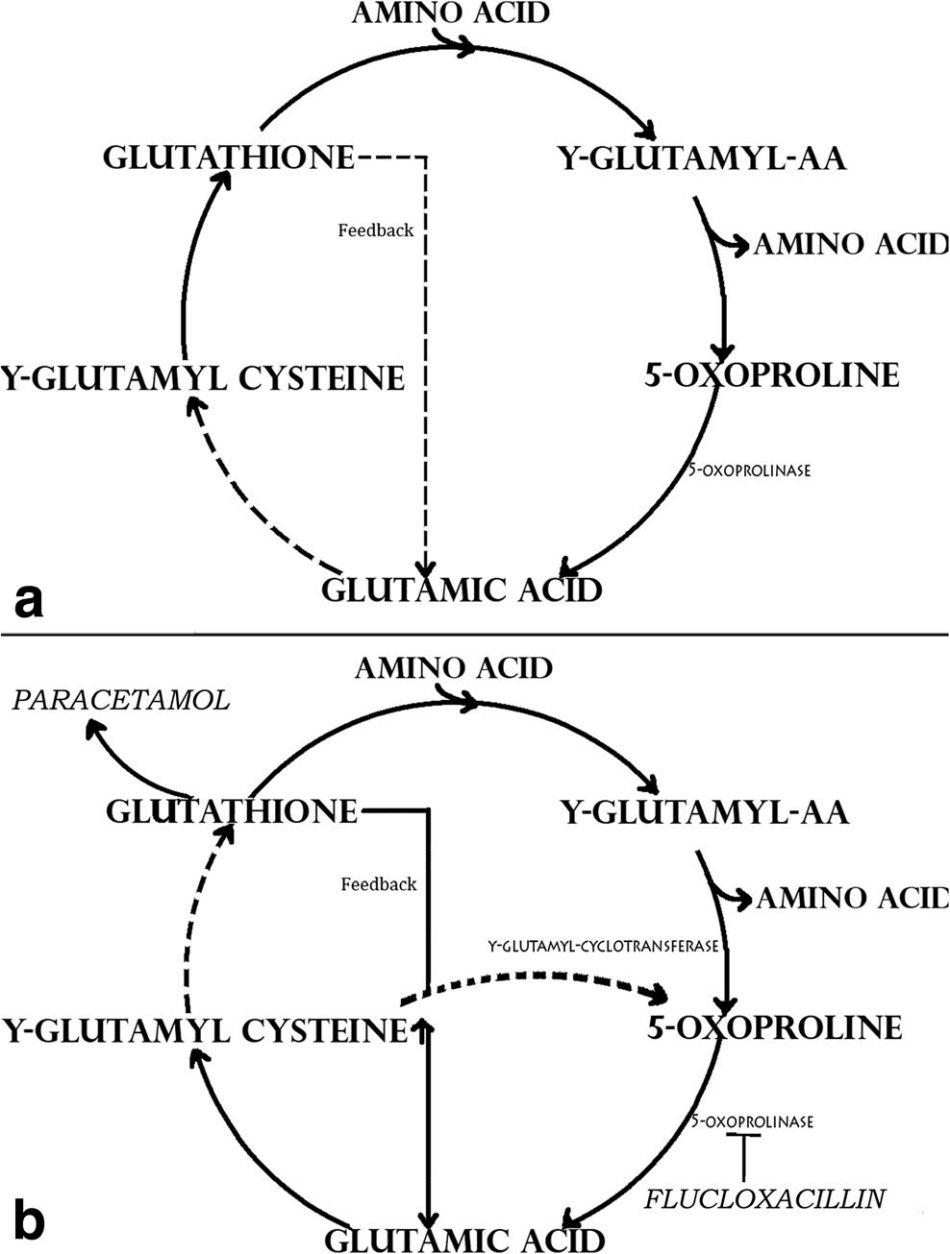
y-Glutamyl cycle (**a**) and effect of paracetamol and flucloxacillin on the y-glutamyl cycle (**b**). **a** In the γ-glutamyl cycle the rate limiting step is the conversion of glutamic acid to γ-glutamyl-cysteine by γ-glutamyl-cysteinesynthetase [8]. Glutathione has a negative feedback on this conversion. In case of lower glutathione levels the formation of glutathione is increased. **b** However, glutathione can be depleted when it is needed intensively, such as in the metabolism of drugs. In the case of paracetamol, the toxic metabolite of paracetamol N-acetylbenzoquinonimine (NAPQI) binds irreversible to glutathione, and perhaps glutathione synthetase, by which glutathione levels can be depleted [2]. Enough y-glutamyl cysteine is produced, but the addition of glycine to y-glutamyl cysteine to form glutathione is too slow to provide for the demand. This is the new rate limiting step (*dashed line*). The intracellulair concentration of γ-glutamyl-cysteine increases and a part of the overproduced y-glutamyl cysteine is converted back to 5-oxoproline (an earlier intermediate) by γ-glutamyl-cyclotransferase (*dashed line*) [8–10]. Flucloxacillin could decrease the activity of 5-oxoprolinase (T form) [3, 9]. This enzyme is involved in the conversion of 5-oxoproline in glutamic acid. A decreased activity can cause accumulation of 5-oxoproline

The amino acid 5-oxoproline is an intermediate of the γ-glutamyl cycle. This cycle is involved in the breakdown of amino acids through an interaction with glutathione (Fig. 1). Glutathione has a negative feedback on γ-glutamyl-cysteinesynthetase. In cases of low glutathione levels, the limited negative feedback ensures sufficient formation of γ-glutamyl-cysteine from which glutathione is formed [8–10]. An excess of 5-oxoproline could be formed in the metabolism of drugs. An example of such drug is paracetamol, which is oxidised to the toxic metabolite N-acetyl-P-benzoquinonimine (NAPQI). NAPQI binds rapidly to glutathione, and may result in lower glutathione levels, especially at toxic dosages or long-term supratherapeutic levels of paracetamol. It is also assumed that NAPQI binds glutathione synthase and inhibits the formation of glutathione [2]. As a consequence of low glutathione and the lack of negative feedback, an increase of γ-glutamyl-cysteine production takes place. The excess γ-glutamyl-cysteine is converted into 5-oxoproline, an earlier intermediate in the cycle [8–10].

Various case reports suggest that the antibiotic flucloxacillin can inhibit the activity of 5-oxoprolinase: the enzyme involved in the conversion of 5-oxoproline in glutamic acid [3, 4, 9]. Decreased activity of 5-oxoprolinase could lead to accumulation of 5-oxoproline, thereby lowering the serum pH by dissociation of the hydrogen ion of the acid group 5-oxoproline.

Theoretically, simultaneous use of paracetamol and flucloxacillin results in an increase of 5-oxoproline by (1) the depletion of glutathione, (2) increased conversion of γ-glutamyl cysteine to 5-oxoproline and (3) decreased conversion of 5-oxoproline to glutamic acid.

As 5-oxoproline is an anion at physiological pH, accumulation of this anion can influence the anion-cation equilibrium between the available anions and cations in the blood. This equilibrium can be calculated with anion and cation levels. Due to the accumulation of 5-oxoproline, the calculated anion gap increases [11, 12].

The combination paracetamol and flucloxacillin is commonly used in hospitals and general practice, and the question arises if this interaction should be checked by automatic alert systems. Whether this would be a proportional safety measure depends on the absolute prevalence of this interaction, which is currently not known.

In this paper, we will discuss a patient with severe metabolic acidosis probably caused by the combination of paracetamol and flucloxacillin. To investigate the relevance of this interaction in clinical practice, we performed a single-centre retrospective study. The following research questions described: (1) How many of the inpatients receive a combination of paracetamol and flucloxacillin? (2) How many of these patients suffer from acidosis during or directly after simultaneous use of these two drugs? (3) How many of the patients receiving both drugs and having an acidosis also have an anion gap? (4) In how many patients with low serum pH were 5-oxoproline values measured and excessively high?

## Case

A 73-year-old male, with a history of haemophilia B, chronic hepatitis B, diabetes type 2 and (presumably hypertensive) chronic kidney disease stage III, was treated for a soft tissue infection with bacteremia caused by *Staphylococcus aureus* following total hip replacement*.* The patient developed progressive tachypnoea, a progressive confusion state and was malnourished, while his other vital signs remained unaffected. On laboratory evaluation there was a partially respiratory compensated metabolic acidosis (pH value of 7.34 (ref. 7.35–7.45); pCO_2_ 2.4 kPa (ref. 4.4–6.3 kPa)). The calculated anion gap at this moment was 19.9 mmol/L (Na^+^ 160 mmol/L (ref.135–145 mmol/L), Cl^−^ 130 mmol/L (ref. 98–107 mmol/L), HCO_3_^−^10.1 mmol/L (ref. 23–29 mmol/L)). As albumin contributes to the anion-cation equilibrium and the albumin level of this patient was low (23 g/L (ref. 35–50 g/L)), the calculated anion gap was corrected to 18.7 mmol/L (ref. 8–16 mmol/L). The anion gap in this patient was high and more unmeasured anions were available in the bloodstream than under normal conditions. All common causes of acidosis, such as diabetic ketoacidosis, lactic acidosis and end-stage renal failure, were not applicable in this situation [12]. Lactate values were within normal limits 0.9–1.0 mmol/L (ref. 0.4–2.0 mmol/L). The possibility of unknown ingested poisons was unlikely, as the patient was already hospitalised for some time and had no access to exogenous substances.

For 14 days, the patient had been receiving 6 g i.v. flucloxacillin per day. Paracetamol 1 g three times daily (tid) was given i.v. initially for pain relieve and was changed to rectal administrations in the same dose. Further treatment consisted of cyclokapron 1000 mg tid; tolbutamide 500 mg twice a day (bid) and haldol droplets bid; alfacalcidol 0.25 μg, thiamine 100 mg, melatonin 3 mg and benefix 6000 IE all once a day (qd); insulin was dosed based on glucose levels. A duodenum tube was placed for additional feeding.

The sodium levels normalised, but he remained confused. Since no other cause for the metabolic acidosis could be found, the possibility of accumulation of 5-oxoproline by paracetamol and flucloxacillin was recognised and the treatment with paracetamol was stopped. The antibiotic treatment of flucloxacillin was switched to ciprofloxacine 400 mg bid and ceftriaxone 2000 mg qd. N-acetylcysteine was preventively started as an antidote of the toxic metabolite NAPQI. Combined with oral administrations of sodium bicarbonate 1000 mg four times daily and a single i.v. administration of 8.4 g, the serum levels HCO_3_^−^ increased rapidly (Supplementary material) and there was a restoration of normo-acidemia.

The concentration of paracetamol level was measured to exclude toxic paracetamol levels. Thirteen hours after discontinuation of the prescription of paracetamol, the paracetamol levels were 2 mg/L (ref. therapeutic concentrations 10–20 mg/L). With the relatively low level of 2 mg/L, it can be concluded that the paracetamol level was not within toxic levels. The malnourished state of the patient could be a contributing factor to low glutathione stores, leading to high 5-oxoprline levels [13]. 5-Oxoproline concentration in the urine of the patient was 10 mmol/mmol creatinine (ref. < 0.1 mmol/mmol creatinine). Shortly after, the patient died from cardio-respiratory failure.

## Methods

To answer the research questions, we performed a retrospective study. We used an earlier collected prescription database of the hospital pharmacy from patients clinically admitted in our tertiary referral centre, the Academic Medical Center, Amsterdam, the Netherlands (AMC) and linked these data to the clinical laboratory data on serum pH, electrolytes (Na^+^, Cl^−^, HCO_3_^−^), albumin and 5-oxoproline measurements.

### Determination of simultaneous use of paracetamol and flucloxacillin

The prescription database of the pharmacy contained data from November 2010 until October 2012. The prescriptions for patients on the intensive care units (ICU), operating rooms (OR) or recovery rooms were not included in this database. From the prescription database of the pharmacy, clinically admitted patients were selected with simultaneous use of paracetamol and flucloxacillin.

Patients were included until the prescription(s) of one of the drugs or both drugs were stopped for more than 24 h. In case of restart of both drugs within 24 h, for example due to relocation within the hospital, a consecutive period of use was indicated. If both drugs were restarted after 24 h, this was indicated as a separate period of simultaneous use for a single patient.

### Identification of patients with acidosis

The patients selected from the pharmacy database were linked to the available laboratory data, by an electronic database search based on patient identification number.

To obtain the prevalence of acidosis patients with a serum pH value of ≤ 7.35, measured within the period of simultaneous use of both drugs until 48 h after one of the drugs had been stopped, were selected. We used 48 h as cut-off value for evaluating the laboratory data, because it is not known how fast the 5-oxoproline levels and the other measured laboratory values change to normal levels after discontinuation of the drugs. Normally after withdrawal, paracetamol and flucloxacillin are no longer within therapeutic plasma concentrations after <24 h. However, the included patients were all hospitalised in a tertiary referral centre, and could have had abnormal liver and kidney functions. In these patients, the drugs could be present longer in the circulation and therefore affect the γ-glutamyl cycle. In order to obtain all available information, we used a longer period (48 h) for evaluating the laboratory data.

Multiple laboratory measurements were available during the period of simultaneous use for a single patient. In order to evaluate the data of all patients in the same way, we used the first available data after start of the treatment of both drugs and for the consecutive days we used the laboratory measurements closest to noon of each day in the period of simultaneous use. This is different compared to clinical practice were each moment of measurement is used, as in the presented case.

### Calculation of anion gap

From the available laboratory data, the anion gap was calculated for the patients with a serum pH ≤ 7.35. The best way to relate the serum pH and calculate the anion gap is to use the data of blood withdrawals taken at one single moment to calculate the anion gap correctly. Over time, small changes in the anion-cation equilibrium are common and can influence the calculated anion gap.

Different calculation methods and reference ranges are possible to determine the anion gap. More information can be found in the supplementary material. The anion gap could be best represented in our centre with the following equation:

$$ \mathrm{Anion}\ \mathrm{gap}={Na}^{+}-{Cl}^{-}-{HCO_3}^{-} $$

The reference range accompanying this calculation is 8–16 mmol/L.

For albumin levels below 35 g/L, the anion gap was adjusted using the following equation:

$$ \mathrm{Adjusted}\kern0.50em \mathrm{anion}\ \mathrm{gap}=\left({Na}^{+}-{Cl}^{-}-{HCO_3}^{-}\right)-\frac{\left(35-\mathrm{Albumin}\right)}{10} $$

### Relation to 5-oxoproline levels

To obtain insight into the measurement and values of 5-oxoproline in patients using both drugs and having low serum pH, an electronic search was conducted on basis of the patient identification number.

### Data analysis

Data of the patients were evaluated and registered anonymously. All procedures performed were in accordance with the ethical standards of the institution and national research committee. The Medical Ethical Committee of the AMC provided a letter of no objection to perform the study as formal consent of patients is not required in the Netherlands for retrospective studies. Data were analysed in SPSS statistics 23 by frequencies and percentages were calculated using cross tabs of nominal values.

## Results

### Simultaneous use of paracetamol and flucloxacillin

**Fig. 2:**
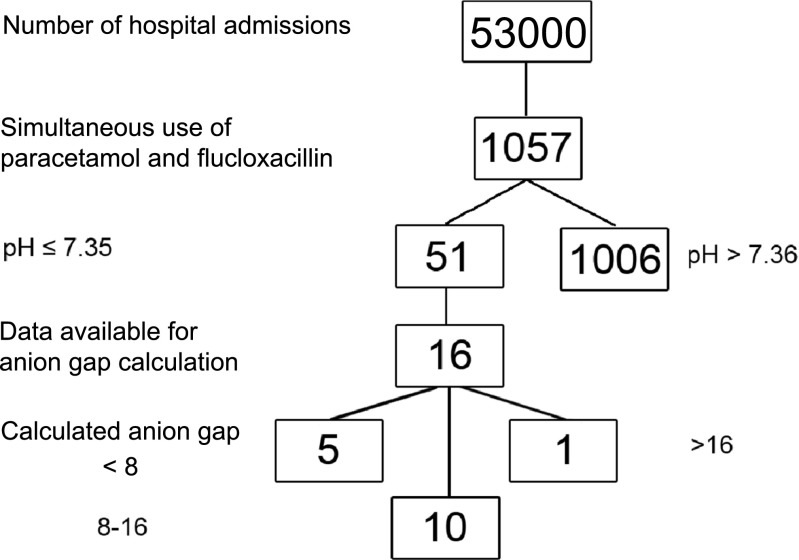
Number and percentages of included patients in the study

In total, 1057 inpatients had a prescription for paracetamol and flucloxacillin at the same time between October 29th 2010 and October 31th 2012 (Fig. 2). In this period, there were approximately 26,500 hospitalizations in our centre each year [14, 15]. Therefore, the percentage of patients using both drugs simultaneously is approximately 2% of all 53,000 hospitalizations.

### Acidosis during simultaneous use

From the 1057 patients, 51 patients (4.8%) had a serum pH value of ≤ 7.35 during the period of simultaneous use, or within 48 h of discontinuation of 1 or both drugs. When the result of simultaneous use of paracetamol and flucloxacillin is included (1057 patients out of 53,000 hospitalizations), approximately 0.1% of all hospitalised patients have risk of having acidosis during the use of both drugs.

From these 51patients, 28 patients had multiple periods of simultaneous use. For 6 patients, the next period of combined use started between 24 and 48 h after discontinuation of 1 or both drugs. The time between the different periods of combined use was between 49 h and a couple of months for the other 22 patients.

Only 12 patients had an acidosis within 48 h after 1 or both drugs were stopped. We assumed that all other patients did not have an acidosis. This includes the patients of whom the serum pH was not measured, as this measurement was not indicated for their treatment.

### Acidosis and anion gap during simultaneous use

Of the 51 patients with low serum pH, 16 patients had sufficient data to calculate the anion gap (Table 1). From the other 35 patients, not all necessary electrolytes were measured in one blood withdrawal; in most cases, the chloride value was missing and therefore the anion gap could not be calculated accurately for these patients.

**Table 1:** Serum concentrations and calculated anion gap of patients with acidosis during and after simultaneous use of paracetamol and flucloxacillin

Subject	pH	Na (mmol/L)	Cl (mmol/L)	HCO3 (mmol/L)	K (mmol/L)	Albumin (g/L)	Anion gap (mmol/L)	Adjusted anion gap (mmol/L)
During simultaneous use								
1	7.26	133↓	107	23.4	3.4↓	22	*2.6*	*1.3*
2	7.35	128↓	106	18.8↓	3.6		*3.2*	NA
3	7.31	136	108	23.3	3.8		*4.7*	NA
4	7.33	149↑	113↑	28.1	4.9↑		*7.9*	NA
5	7.29	135	94↓	31.5↑	4.4		*9.5*	NA
6	7.31	131↓	103	18.2↓	4.6↑	34	*9.8*	*9.7*
7	7.33	153↑	122↑	12.6↓	3.4↓		*18.4*	NA
Within 48 h after simultaneous use								
8	7.34	133↓	106	23.1	4.5		*3.9*	NA
9	7.23	127↓	106	15↓	3.8		*6*	NA
10	7.34	137	111↑	18.7↓	4.3		*7.3*	NA
11	7.35	143	106	28.2	3.9		*8.8*	NA
12	7.19	132↓	102	21↓	3.8		*9*	NA
13	7.34	151↑	118↑	23.7	2.6↓		*9.3*	NA
14	7.26	138	106	19.6↓	4.6↑		*12.4*	NA
15	7.25	138	109↑	16.3↓	6.3↑		*12.7*	NA
16	7.3	134↓	107	12↓	5.9↑		*15*	NA

Arrow down: measured electrolyte below reference range of electrolyte. Arrow up: measured electrolyte above reference range of electrolyte. Reference ranges: Na (sodium): 135–145 mmol/L, Cl (chloride) 98–107 mmol/L, HCO3: bicarbonate 23–29 mmol/L, K (), 3.5–4.5 mmol/L

*NA* not available

Five out of 16 patients showed an anion gap below the reference range of 8–16 mmol/L during simultaneous use or within 48 h after withdrawal of one of the drugs. Only one patient had an anion gap above the reference range namely, 18.4 mmol/L (ref. 8–16 mmol/L). This was the same patient as presented in the case. When combined with the admission data this would give a prevalence of approximately 0.002% of all admitted patients. For ten patients, the calculated anion gap was within the reference range.

### Acidosis and high 5-oxoproline values

Of only 2 out of the 51 patients with a low serum pH, organic acids in urine were measured. Only one patient had high urine level of 5-oxoproline. This was the same patient as presented in the case report. The 5-oxoproline levels of the second patient, numbered 1 in Table 1, were below the reference of < 0.1 mmol/mmol creatinine.

## Discussion

We presented a patient with severe metabolic acidosis. Simultaneous use of paracetamol and flucloxacillin was probably the cause of the acidosis, as the 5-oxoproline concentration in urine was significantly increased to the toxic level of 10 mmol/mmol creatinine (reference <0.1 mmol/mmol creatinine) and all common causes for acidosis were not applicable.

As severe acidosis is potentially life threatening, we investigated the prevalence of the paracetamol and flucloxacillin interaction in relation to acidosis, high anion gap and increased 5-oxoproline levels in urine in clinically admitted patients in our tertiary hospital.

We limited the scope of this study to patients with measured pH values ≤ 7.35, thereby excluding patients with fully respiratory compensated metabolic acidosis. In these patients, the acidosis was probably less severe and the patient could cope with the acidosis by other mechanisms. As measurement of serum pH is often performed after HCO_3_^−^ measurements, we only included the patients with more severe acidosis and possibly patients with unrecognised acidosis.

In this retrospective study, we identified only one patient with a high anion gap metabolic acidosis. This is the same patient as presented in the case (subject 7 in Table 1). From 1057 patients using both drugs, this single patient with high 5-oxoproline levels, probably due to this drug interaction was identified in clinical practice. Therefore, we can conclude that the current lack of surveillance policy in regard to this interaction is acceptable. However, the severity could possibly be decreased if the interaction was recognised earlier.

The retrospective design of this study is a limitation and may have led to underestimation of the reported prevalence numbers. Four issues could be identified: (1) We had only data available collected from regular patient care. Although unlikely, we may have missed patients, because measurements such as serum pH or certain electrolytes were not always indicated for the treatment of the patients. (2) We used a prescription database instead of data on the actual administration of paracetamol and flucloxacillin to the patients. Especially for a drug as paracetamol, which is often prescribed for use on request on basis of pain, the absence of administration information is a major limitation. (3) The drugs prescribed on ICU, OR and recovery rooms were not included in the pharmacy database. Patients receiving paracetamol and flucloxacillin exclusively at these units were not included. However, patients at these units often receive other pain medication such as opioids instead of paracetamol. (4) The 53,000 admitted patients in the hospital could include multiple admissions of a single patient. In contrast to the admission number, patients included in this study were only calculated once.

From the data set, it became clear that pH measurements and individual electrolytes are not always measured from the same blood withdrawal in normal patient care. Therefore, accurate calculations of the anion gap were not possible for all patients with acidosis. Furthermore, compared to the other electrolytes, chloride was often lacking or was taken at an earlier or later time point. Small changes could already influence the calculated anion gap by some points.

Five out of 51 patients with acidosis in this study had an anion gap below the reference range. Theoretically, one expects a high anion gap due to increased levels of the anion 5-oxoproline. Therefore, the acidosis measured in the patients with a low anion gap was probably caused by factors other than 5-oxoproline. More information about this subject can be found in the supplementary material.

Regarding generalizability of our data, we remark the following. Despite the use of an older database (Nov. 2010-Oct. 2012), the prevalence of high anion gap metabolic acidosis caused by simultaneous use of paracetamol and flucloxacillin has probably not changed as both drugs are commonly used in patient care and the prescribing policy has not changed in the Netherlands over recent years. We only included patients admitted into a tertiary referral centre resulting in including patients with more risk factors for developing acidosis. In case reports, several other risk factors for acidosis are mentioned, such as female gender, age, chronic alcoholism, liver and end-stage renal dysfunction, sepsis and malnutrition [7–9, 16, 17].

Automatic alert systems based only on simultaneous use of paracetamol and flucloxacillin is not supported by the very low risk of metabolic acidosis we found during the combined use of paracetamol and flucloxacillin and the identification of the only patient with metabolic acidosis during clinical practice. As both drugs are used frequently in hospitals and general practice, a lot of unnecessary signals would be created and contribute to alert fatigue. Alert systems based on toxic paracetamol levels are not representative, as the patient presented in the case had a subtherapeutic paracetamol level. The development of a clinical rule, in which physicians and pharmacists are alerted on basis of combined pharmacy, laboratory data and clinical data, could be a good alternative. This clinical rule should be based on simultaneous use of paracetamol and flucloxacillin and pH value ≤ 7.35. Based on the data of the current study only for 25 patients alerts will be generated each year. This would probably not increase the risk of alert fatigue but can alert the physicians and pharmacists for possible harm to a patient earlier.

After receiving an alert on basis of the clinical rule, we advise to measure all the necessary electrolytes, including chloride, for calculating the anion gap and narrowing the differential diagnosis. A high anion gap is a strong indication towards a 5-oxoproline accumulation when paracetamol and flucloxacillin are used simultaneously. Measurement of 5-oxoproline is directly indicated to identify the interaction with certainty and have insight into the severity of the accumulation. At this moment, the measurement of 5-oxoproline is not always available and is time consuming.

To improve the selection of patients with the clinical rule and create less unnecessary alerts, prospective research into risk factors such as malnutrition, sepsis, liver and renal failure is needed. This prospective study could be performed on basis of the proposed clinical rule. In this study, the scope could be extended to patients with acidosis and respiratory compensation by selecting patients on low HCO_3_^−^ levels first and sequentially on low pH values. Additionally, it is recommended to use information from the patient on drug use at home, the actual administration data of both drugs and to measure the 5-oxoproline values in order to obtain a clear relationship between the acidosis and the simultaneous use of both drugs.

## Conclusion

Our data indicate that the prevalence of clinical manifestations of high anion gap metabolic acidosis is very low in patients using paracetamol and flucloxacillin simultaneously. The only patient identified was the same patient presented in the case report. On basis of these data and the identification of this patient during normal patient care, we can conclude that the current surveillance policy is correct. Interaction warning systems on basis of prescription data alone should not be implemented, due to the low prevalence and the risk of alert fatigue.

However, a clinical rule combining data of paracetamol, flucloxacillin prescriptions and pH values could be implemented in order to recognise these patients earlier and treat them effectively to reduce harm. When alerted by acidosis and combined drug use, all necessary electrolytes, including chloride, needs to be measured in order to calculate the anion gap and to narrow the differential diagnosis. Also, measurement of 5-oxoproline levels in blood or urine is indicated to identify the interaction of these two drugs with certainty and to have insight into the severity of the accumulation. These steps are needed to be taken even in case of subtherapeutic levels of paracetamol and/or flucloxacillin.

## Electronic supplementary material

ESM 1 (DOC 67 kb)
