# Supplementary material for: Metabolic acidosis caused by concomitant use of paracetamol (acetaminophen) and flucloxacillin? A case report and a retrospective study
Source: Eur J Clin Pharmacol. 2017 Aug 7;73(11):1459–65. doi: 10.1007/s00228-017-2311-6 (PMC5662679; doi:10.1007/s00228-017-2311-6)
Supplement: Supplementary file 1 — (DOC 67 kb) [file 228_2017_2311_MOESM1_ESM.doc]

# Supplementary material

### Bicarbonate levels over time:

**
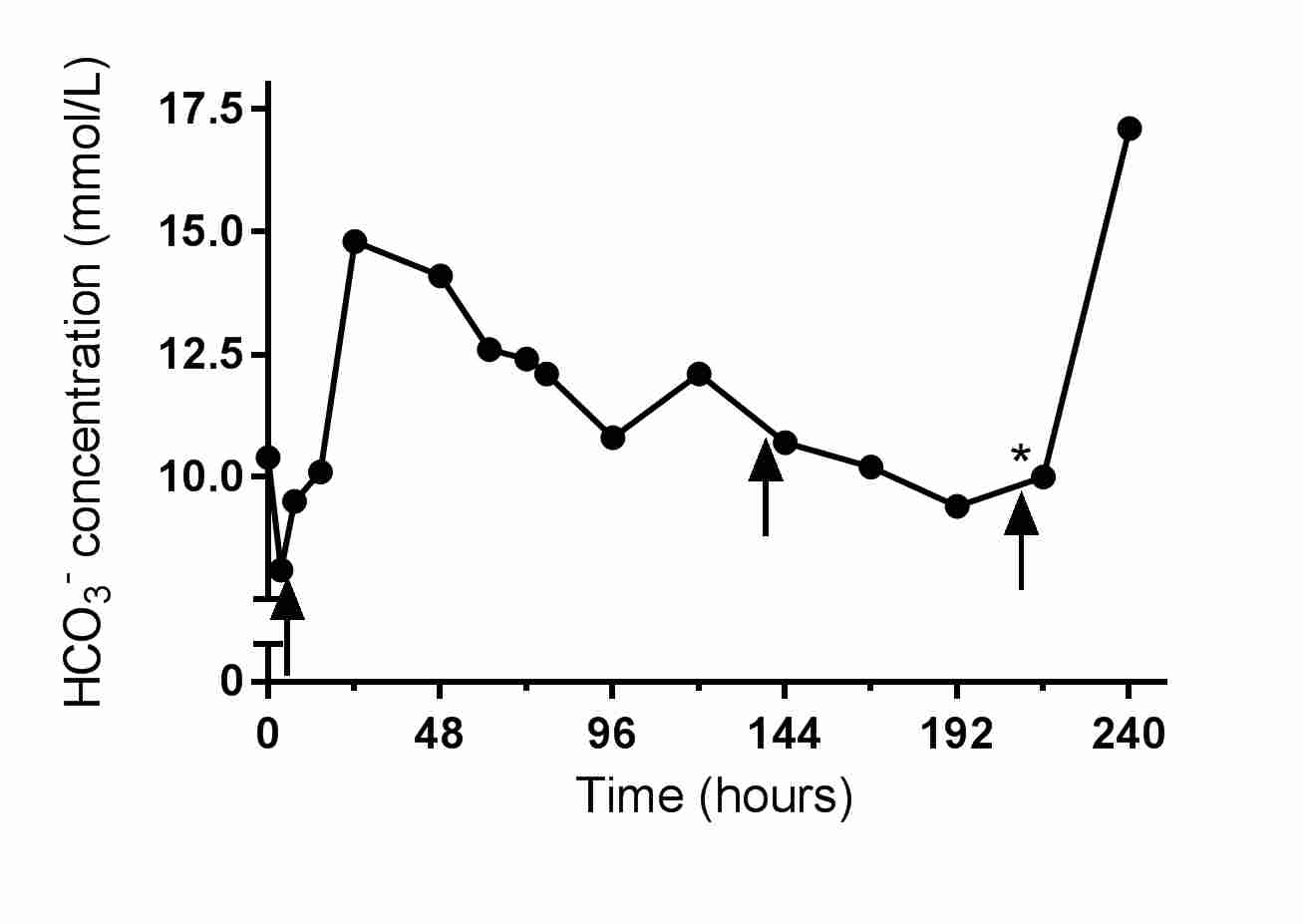
**

**Fig: Bicarbonate levels over time.** The arrows represent infusion of bicarbonate intravenously and the asterisk represents the discontinuation of paracetamol.

### Detailed considerations in regard to the anion gap calculation:

The methods to calculate the anion gap can vary among laboratories, depending on the analytical analyses of each individual electrolyte. Therefore, each laboratory determines its own reference range in conjunction with its method of calculation [1-3].

In our hospital laboratory the following equipment was used for measuring the different electrolytes. Sodium and potassium were either measured in heparin plasma on the ISE-900 unit of the Modular system or the Cobas 8000 (Roche Diagnostics) or in heparinised whole blood on a RapidLab 865 or 1265 blood gas apparatus (Siemens).

Bicarbonate was measured in heparin plasma on a P-800 (Modular system) or a c502 (Cobas 8000 system) analytical module or calculated from pH and pCO2 measurements in heparinised whole blood on the blood gas equipment.

The calculation used in this study was determined by an earlier validation study by our hospital laboratory.

In the current study multiple patients had a low anion gap. Low anion gap is relatively rare compared to high anion gap. Causes or errors leading to low or even negative anion gaps are recently discussed by M. Emmet and include, random laboratory inaccuracies, hypoalbuminemia, hypercalcemia, lithium toxicity, pseudo-hyponatremia due to displacement errors, hyper viscosity of plasma or serum, or hyperlipidemia [2]. Due to the high number of patients with a low anion gap in our study, a scan was performed by our laboratory on 1,500 samples collected from the overall hospital population in the period of our collected data. The median anion gap was circa 10.3 mmol/L. The point estimate of the anion gap of the patients included in the presented study is 8.9 mmol/L (95%CI: 6.5 -11.1 mmol/L) (table 1). As this is not significantly different, we can’t conclude that the anion gap in our study population is different compared to the overall hospital population.

**References**

1. Kraut JA, Madias NE (2007) Serum anion gap: its uses and limitations in clinical medicine. Clinical journal of the American Society of Nephrology : CJASN 2 (1):162-174. doi:10.2215/cjn.03020906

2. Emmett M (2015) Approach to the Patient With a Negative Anion Gap. American journal of kidney diseases : the official journal of the National Kidney Foundation. doi:10.1053/j.ajkd.2015.07.024

3. Winter SD, Pearson JR, Gabow PA, Schultz AL, Lepoff RB (1990) The fall of the serum anion gap. Archives of internal medicine 150 (2):311-313
